# Chymase-Cre; Mcl-1^fl/fl^ Mice Exhibit Reduced Numbers of Mucosal Mast Cells

**DOI:** 10.3389/fimmu.2019.02399

**Published:** 2019-10-15

**Authors:** Ying Luo, Nicole Meyer, Qingqing Jiao, Jörg Scheffel, Carolin Zimmermann, Martin Metz, Ana Zenclussen, Marcus Maurer, Frank Siebenhaar

**Affiliations:** ^1^Dermatological Allrgology, Department of Dermatology and Allergy, Charité - Universitätsmedizin Berlin, Corporate Member of Freie Universität Berlin, Humboldt - Universität zu Berlin, and Berlin Institute of Health, Berlin, Germany; ^2^Experimental Obstetrics and Gynecology, Medical Faculty, Otto-Von-Guericke-University, Magdeburg, Germany

**Keywords:** mast cells, mouse model, mucosa, connective tissue, chymase, Cre, Mcl-1

## Abstract

Mast cells (MCs) are considered as key effector cells in the elicitation of allergic symptoms, and they are essential players in innate and adaptive immune responses. In mice, two main types of MCs have been described: connective tissue MCs (CTMCs) and mucosal MCs (MMCs). However, little is known about the biological functions of MMCs, which is due to the lack of suitable models to investigate MMCs *in vivo*. Here, we aimed to generate a mouse model selectively deficient in MMCs. It has been previously described that Cre expressed under the control of the baboon α-chymase promotor is predominantly localized in MMCs. Therefore, we mated α-chymase-Cre transgenic mice with mice bearing a floxed allele of the myeloid cell leukemia sequence 1 (Mcl-1). Mcl-1 encodes for an intracellular antiapoptotic factor in MCs; hence, a selective reduction in MMCs was expected. Our results show that this new mouse model contains markedly reduced numbers of MMCs in mucosal tissues, whereas numbers of CTMCs are normal. Thus, Chm-Cre; Mcl-1^fl/fl^ mice are a useful tool for the investigation of the pathophysiological functions of MMCs *in vivo*.

## Introduction

Mast cells (MCs) are potent inflammatory cells that are constitutively present in most tissues. MCs are key effector cells in the elicitation of allergic symptoms (1–3) and essential players in protective innate and adaptive immune responses to pathogens and other environmental threats (4–6).

MCs exhibit a diverse hematopoietic origin. As the bone marrow was previously believed the only site from which MCs can arise (7–9), it has been recently shown that MCs also originate from the yolk sac and from definite progenitors (10). Unlike other myeloid-derived cells, which differentiate and mature in the BM before being released to the blood, MCs egress the BM and circulate as immature progenitor cells (11–14) (generically termed MCps), which give rise to mature MCs when they migrate to their target tissues (15).

In mice, MCps differentiate into two major types of mature MCs, connective tissue MCs (CTMCs) and mucosal MCs (MMCs), classified according to their anatomical distribution: CTMCs are found in skin, peritoneum, and submucosa of the gastrointestinal tract, where they are predominantly located in close proximity to vessels and sensory nerve endings. In contrast, MMCs are present in mucosal tissues such as the gastrointestinal and respiratory mucosa as well as in the uterus where they coexist with CTMCs and an intermediate phenotype (15, 16). CTMCs and MMCs also differ in their morphology, protease expression profiles, and biochemical and functional properties (15). CTMCs represent a robust and long-lived tissue population (17, 18), whereas MMCs are low in numbers under physiological conditions but show rapid and marked expansion under pathological conditions, such as parasitic infections (19–21) or food allergy (22). In addition, it has been reported that MMCs can expand during T cell-dependent immune responses (19, 23, 24), whereas CTMCs exhibit little or no T cell-dependent behavior and appear in athymic nude mice or rats in normal numbers. CTMCs are relatively well-characterized and studied as compared to MMCs. This is, in part, because animal models for the investigation of CTMC functions became available some decades ago. In contrast, very little is known about MMCs, and we are lacking suitable models to study their biological functions.

The role of MMCs in health and disease is largely unknown. In contrast to CTMCs, very little is known about the pathways of MMC activation, their physiological functions, mechanisms of proliferation and survival, as well as modulators of MMCs biology. We, therefore, aimed to generate a new mouse model that exhibits a specific reduction in MMCs, thus allowing for the investigation of MMC biology *in vivo*. To this end, we made use of the Cre/loxP recombination system for generating tissue-specific gene inactivation in mice (25, 26). It has been previously reported that Cre expression driven by the baboon α-chymase promotor correlates to MC-specific lineages present in colon and lungs, thereby suggesting an MMC-specific expression (27). Hence, in the present study, we mated chymase-Cre transgenic mice with mice bearing a floxed allele of the myeloid cell leukemia sequence 1 (Mcl-1), which encodes for an intracellular antiapoptotic factor in MCs (28). We hypothesized that the genetic inactivation of Mcl-1 under the control of the α-chymase promotor in this Chm-Cre; Mcl-1^fl/fl^ mouse induces apoptosis in the target cell population and results in a specific reduction of MMCs.

## Result

### Chm-Cre; Mcl-1^fl/fl^ Mice Have Markedly Reduced Numbers of Gastric and Duodenal MMCs

**Figure 1:**
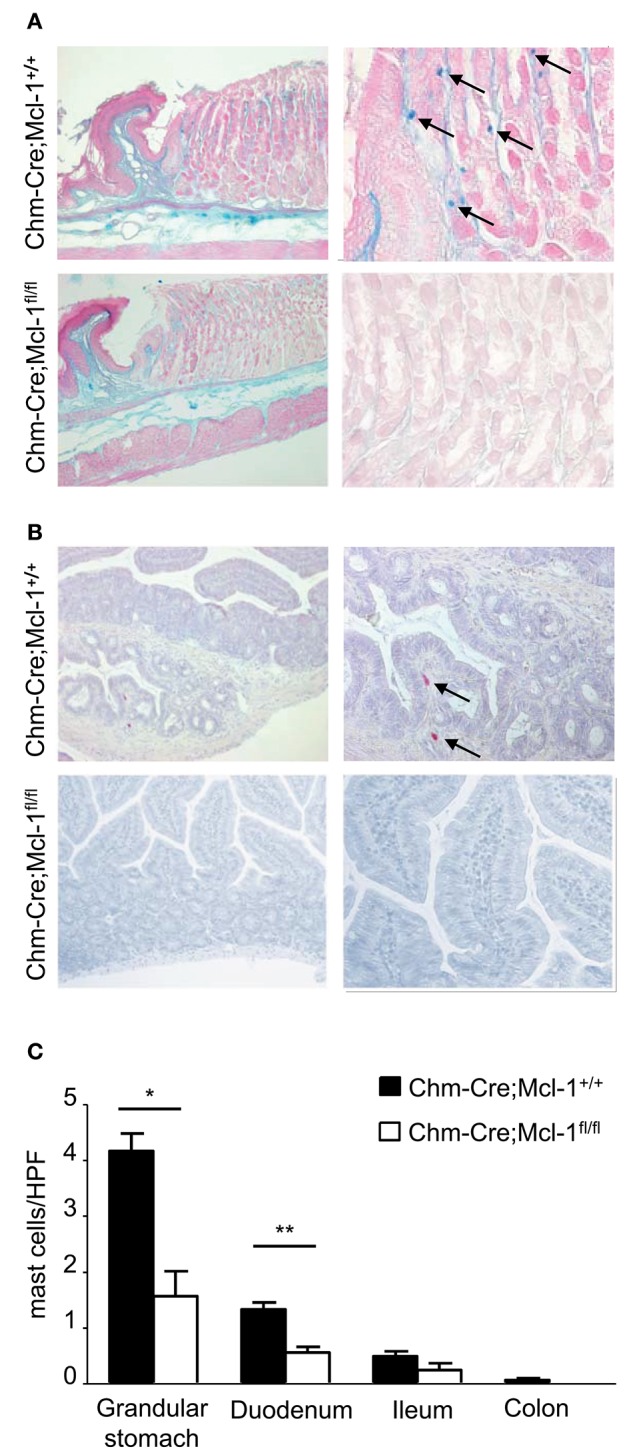
Chm-Cre; Mcl-1^fl/fl^ mice have markedly reduced numbers of representative mucosal mast cell (MMC) populations. **(A)** Alcian blue staining for stomach MCs of 5-μm-thick paraffin sections showed markedly reduced MCs numbers (blue) in Chm-Cre; Mcl-1fl/fl mice compared to control Chm-Cre; Mcl-1^+/+^ mice. **(B)** Chloroacetate esterase staining for intestinal MCs showed decreased number of MCs (red) in duodenum of Chm-Cre; Mcl-1^fl/fl^ mice compared to control Chm-Cre; Mcl-1^+/+^ mice. **(C)** Numbers of MCs in different gastrointestinal tissues were assessed by quantitative histomorphometry analysis. **(A,B)** left: 100× magnification, **(A)** right: 400× magnification, **(B)** right: 200× magnification. Data were pooled from three independent experiments (*n* = 5 mice per group) and expressed as mean ± SEM (*P < 0.05, **P < 0.01, n.s., not significant).

Chm-Cre; Mcl-1^fl/fl^ mice showed markedly reduced numbers of MMCs in the glandular stomach as compared to control Chm-Cre; Mcl-1^+/+^ mice (Figure 1, 1.6 ± 0.5 MCs/HPF vs. 4.12 ± 0.3/HPF). MMC numbers were also markedly reduced in the lamina propria of the duodenum of Chm-Cre; Mcl1^fl/fl^ mice (Chm-Cre;Mcl-1^fl/fl^: 0.6 ± 0.1 MC/HPF vs. Chm-Cre;Mcl-1^+/+^: 1.3 ± 0.1 MC/HPF, −54%, *P* < 0.01). MCs in ileum and colon generally appear in very low numbers; hence, the reduction of MMCs in the lamina propria of the ileum and colon of Chm-Cre; Mcl-1^fl/fl^ mice was detectable but not significant.

### Chm-Cre; Mcl-1^fl/fl^ Mice Exhibit Markedly Reduced Numbers of Uterus MCs and Decreased Placental Thickness

**Figure 2:**
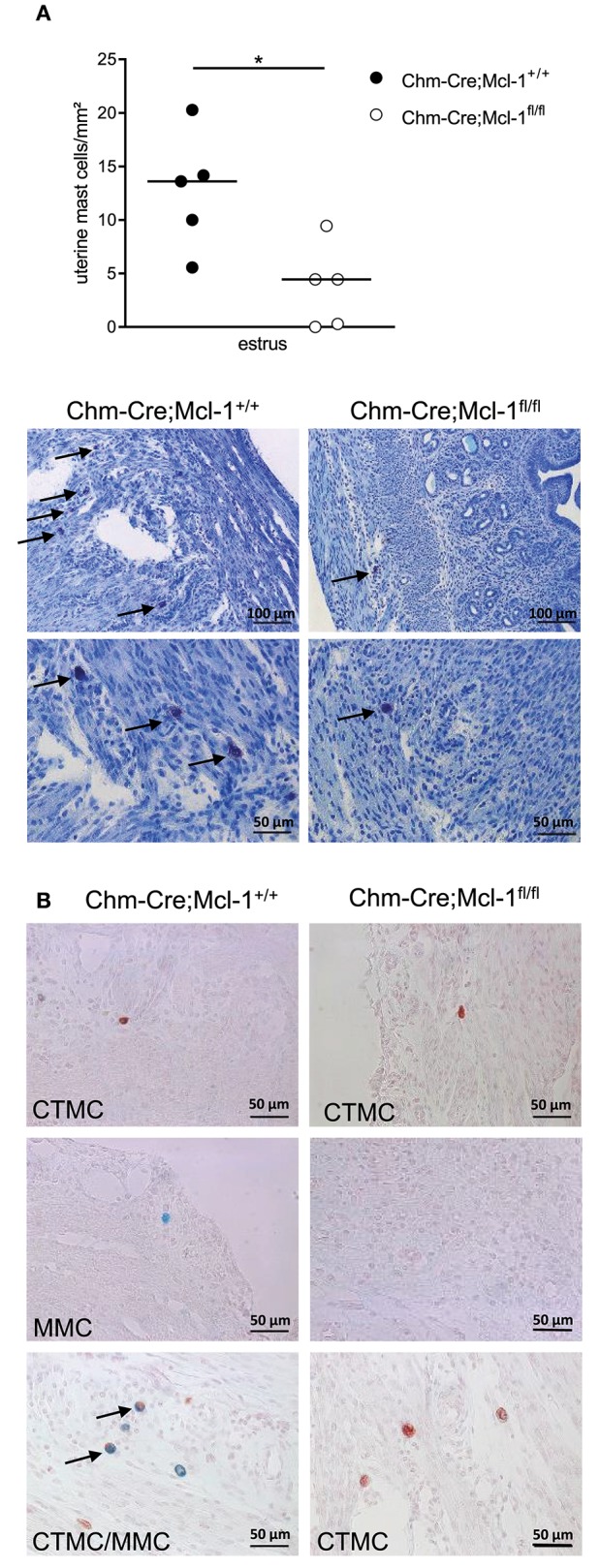
Chm-Cre; Mcl-1^fl/fl^ mice exhibit reduced numbers of uterus MCs. **(A)** Toluidine blue staining of 5-μm-thick paraffin uteri sections showed markedly reduced number of uterus MCs (uMCs) at the estrus cycle (arrows) in Chm-Cre; Mcl-1^fl/fl^ mice compared to control Chm-Cre; Mcl-1^+/+^ mice. **(B)** Representative images of alcian blue (MMCs) and safranin (CTMCs) staining of uterus from Chm-Cre; Mcl-1^+/+^ and Chm-Cre; Mcl-1^fl/fl^ at estrus. Results are presented as individual values and median. Statistical differences were obtained by using Mann–Whitney (*P < 0.05), 200× magnification.

In consideration of the variation of uterine MC numbers (uMCs) during the fertile period in the uterus, which contains MMCs and CTMCs, we quantified the number of uMCs/mm^2^ in the uterus of virgin Chm-Cre; Mcl-1^fl/fl^ and Chm-Cre; Mcl-1^+/+^ female mice at the estrus. During the estrus cycle, Chm-Cre; Mcl-1^fl/fl^ mice presented significantly reduced uMC numbers as compared to Chm-Cre; Mcl-1^+/+^ mice (Figure 2A, 3.72 ± 1.72/mm^2^, *n* = 5 vs. 12.72 ± 2.44/mm^2^, *n* = 5, *P* = 0.017). Histomorphological analyses of uterine sections stained with alcian blue and safranin, to quantify MMCs and CTMCs, respectively, identified both CTMCs and MMCs during estrus in Chm-Cre; Mcl-1^+/+^ control mice. Interestingly, we observed some alcian blue/safranin double-positive cells in the uterus of Chm-Cre; Mcl-1^+/+^ mice, suggesting for an indistinct potentially intermediate phenotype. In contrast, Chm-Cre; Mcl-1^fl/fl^ mice had CTMCs only, but no MMCs (Figure 2B).

**Figure 3:**
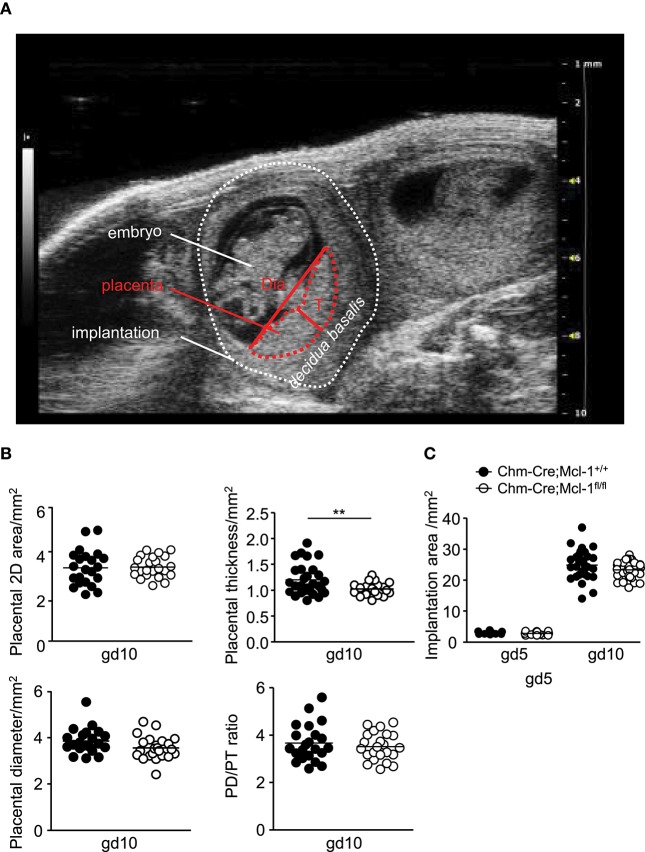
Chm-Cre; Mcl-1^fl/fl^ mice presented decreased placental thickness at gd10. **(A)** Representative ultrasound image of an implantation at gd10 showing the embryo, the *decidua basalis*, and the placenta with placenta thickness (T) and diameter (Dia). **(B)** Placental area, placental thickness, placental diameter, and placental diameter/thickness ratio from Balb/c-paired Chm-Cre; Mcl-1^+/+^ (mice *n* = 4, placentas *n* = 22) and Chm-Cre; Mcl-1^fl/fl^ mice (mice *n* = 5, placentas *n* = 23) at gd10. **(C)** Implantation areas in mm^2^ from Balb/c-paired Chm-Cre; Mcl-1^+/+^ (mice *n* = 4, implantations *n* = 15–32 per day) and Chm-Cre; Mcl-1^fl/fl^ females (mice *n* = 5, implantations *n* = 21–36 per day) at gd5 and gd10. Results are presented as individual values for each single placenta with mean. Statistical differences were obtained using unpaired *t*-test (**P < 0.01). gd, gestation day; T, thickness; Dia, diameter.

To investigate whether the lack of MMCs in the uterus has an impact on fetal/placental growth, we performed ultrasound analyses of the gestation period at gd5 and gd10 assessing the implantation area, placental thickness, and diameter, as well as the placental diameter/thickness ratio of Balb/c-paired Chm-Cre; Mcl-1^fl/fl^ mice (*n* = 5, placentas *n* = 23) and Chm-Cre; Mcl-1^+/+^ mice (*n* = 4, placentas *n* = 22) at gd10 (Figures 3A,B). We observed significantly reduced placental thickness in Chm-Cre; Mcl-1^fl/fl^ mice (Figure 3B), whereas the implantation area, placenta weight, as well as implantation and abortion rates were comparable to the one observed for Chm-Cre; Mcl-1^+/+^ mice at gd5 and gd10 (Figure 3C and Figures S1A–C). Also, no differences in spiral artery (SA) parameters were found at gd10 (Figure S1D).

### Chm-Cre; Mcl-1^**fl/fl**^ Mice Exhibit No Difference in Cell Numbers or Morphology of Representative CTMC Populations

**Figure 4:**
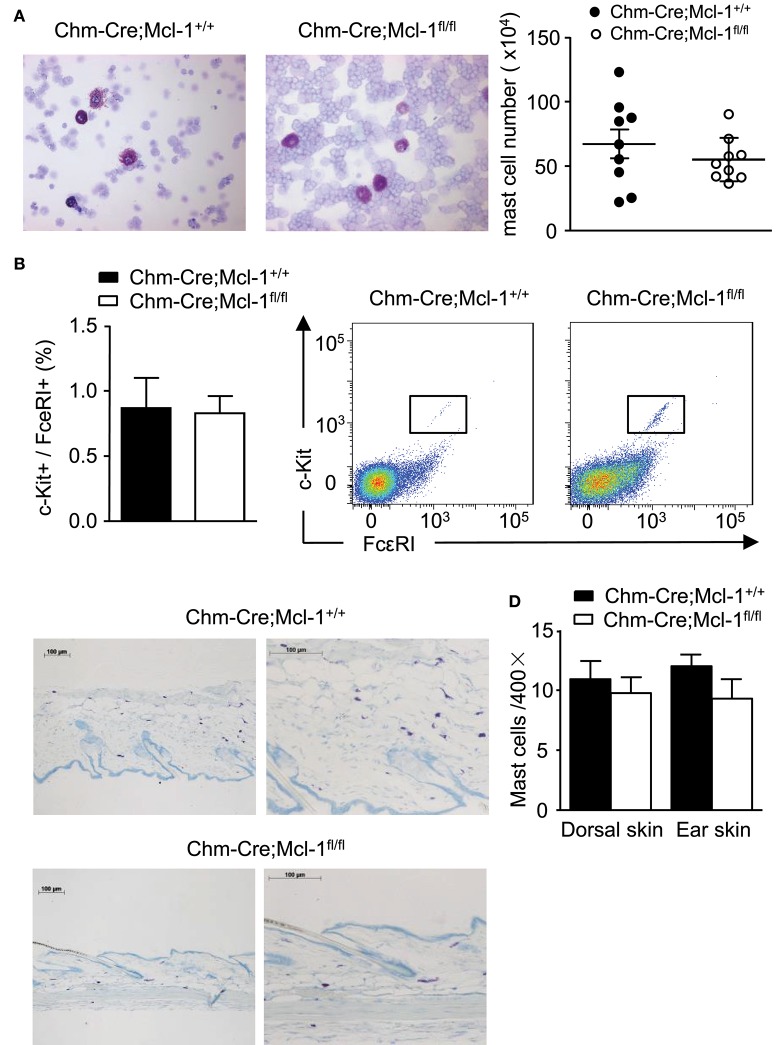
Chm-Cre; Mcl-1^fl/fl^ mice exhibit no variation of cell number in representative sites of connective tissue mast cell (CTMC) populations as compared to Chm-Cre; Mcl-1^+/+^ mice. **(A)** Cytospins of peritoneal lavage fluid from Chm-Cre; Mcl-1^+/+^ mice and Chm-Cre; Mcl-1^fl/fl^ mice were stained with Giemsa solution and MC numbers were assessed by Neubauer Hemocytometry. Mice exhibit comparable numbers of PMCs (right) with similar morphological features. **(B)** Percentage of mast cell surface markers expression (left) and representative flow cytometry plots (right) showed comparable expression of c-kit and FcεRI on PMCs isolated from Chm-Cre; Mcl-1^+/+^ mice and Chm-Cre; Mcl-1^fl/fl^ mice. **(C)** Giemsa staining of 5-μm-thick paraffin sections of dorsal skin obtained from Chm-Cre; Mcl-1^+/+^ and Chm-Cre; Mcl-1^fl/fl^ mice show comparable numbers of MCs (purple). **(D)** MCs in dorsal and ear skin tissues show similar amounts of dermal MCs. **(A)** 400× magnification, **(C)** left: 100× magnification, **(C)** right: 200× magnification. Data were pooled from two (**A,B**; *n* = 3 mice per group ± SEM) or five independent experiment (**C,D**; *n* = 5 mice per group ± SEM).

Chm-Cre; Mcl-1^fl/fl^ mice and Chm-Cre; Mcl-1^+/+^ mice were similar in their numbers of CTMCs obtained from the peritoneum (PMCs), and their PMCs were similar in their morphology and surface expression of c-kit and FcεRI as assessed by FACS analysis (Figures 4A,B). Numbers of CTMCs in the dorsal skin of Chm-Cre; Mcl-1^fl/fl^ and Chm-Cre; Mcl-1^+/+^ control mice were similar as assessed by quantitative histomorphometry (Figures 4C,D; Chm-Cre; Mcl-1^fl/fl^: 10.1 ± 0.9 MCs/HPF; Chm-Cre; Mcl-1^+/+^: 10,6 ± 0.8 MCs/HPF). Both strains also exhibited similar numbers of ear skin CTMCs (Figure 4D; Chm-Cre; Mcl-1^fl/fl^: 11.2 ± 0.7 MCs/HPF; Chm-Cre; Mcl-1^+/+^: 10.1 ± 0.9 MCs/HPF).

### Bone Marrow-Derived Cultured MCs of Chm-Cre; Mcl-1^fl/fl^ Mice-Exhibit Normal Proliferation and Differentiation

**Figure 5:**
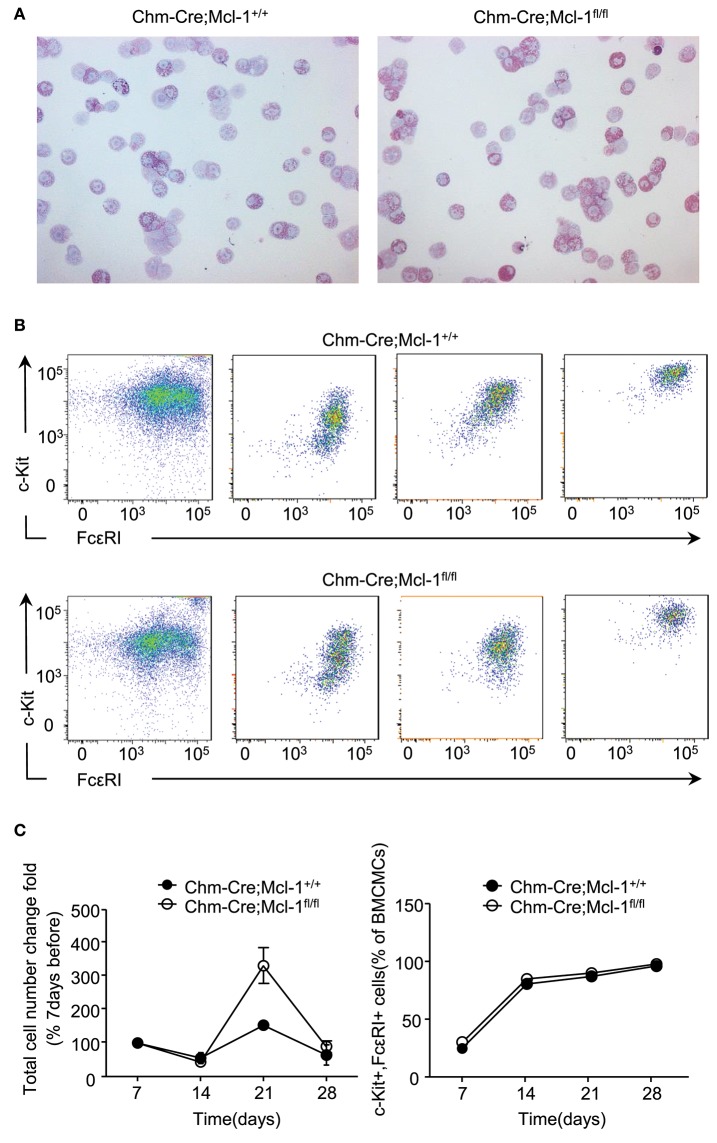
Comparable proliferation and differentiation rates in bone marrow-derived cultured mast cells (BMCMCs). **(A)** Representative light micrograph shows comparable morphology of BMCMCs after 4 weeks of culture in IL-3-supplemented medium from Chm-Cre; Mcl-1^+/+^ control mice and Chm-Cre; Mcl-1^fl/fl^ mice. BMCMCs were identified morphologically on cytospin cell preparations stained with Giemsa solution. **(B)** Representative flow cytometry plots and percentage of c-kit and FcεRI expression show comparable differentiation rate on BMCMCs isolated from Chm-Cre; Mcl-1^+/+^ mice and Chm-Cre;Mcl-1^fl/fl^ mice after 7, 14, 21, and 28 days of culture. **(C)** The proliferation rate of BMCMCs was evaluated by calculating the fold change of cell number over time. Data show similar proliferation rates of BMCMCs after 7, 14, 21, and 28 days of culture from Chm-Cre; Mcl-1^+/+^ mice and Chm-Cre; Mcl-1^fl/fl^ mice. **(A)** 200× magnification. Data are pooled from three independent experiments (*n* = 3 mice per group ± SEM).

It has been previously reported that the α-chymase promotor is not expressed in bone marrow-derived cultured MCs (BMCMCs). As expected, cytological analyses showed cytoplasmic Giemsa-positive granules in both, BMCMCs generated from Chm-Cre; Mcl-1^fl/fl^ and Chm-Cre; Mcl-1^+/+^ mice, after 4 weeks of culture. Furthermore, BMCMCs derived from Chm-Cre; Mcl-1^fl/fl^ and Chm-Cre; Mcl-1^+/+^ mice were similar in size, granule distribution, and nucleus formation (Figure 5A). Chm-Cre; Mcl-1^fl/fl^ and Chm-Cre; Mcl-1^+/+^ BMCMCs also showed no differences in their rates of proliferation after 7, 14, 21, or 28 days of culture (Figure 5B). The differentiation of BMCMCs derived from Chm-Cre; Mcl-1^fl/fl^ and Chm-Cre; Mcl-1^+/+^ was also similar as assessed by flow cytometric analysis of the expression of the MC surface markers CD117 (c-kit) and FcεRIα at day 7, 14, 21, or 28 of culture. BMCMCs from both strains, after 28 days of culture, exhibited more than 95% double-positive cells (Figure 5C).

## Discussion

In this study, we developed a new mouse strain Chm-Cre; Mcl-1^fll/fl^ that was used for the investigation and characterization of different populations of MCs (CTMCs and MMCs). Our data support the conclusion that Chm-Cre; Mcl-1^fl/fl^ mice have markedly reduced numbers of MMCs in mucosal tissues of the glandular stomach and intestine, which contain only MMCs, as well as in the uterus, which contains both subtypes of MCs. In contrast, the numbers of CTMCs, in the dorsal skin, ear skin, and peritoneal cavity, are normal.

MMCs are found at relatively low numbers in most mucosal tissues, especially in the intestines (29). It was, therefore, not surprising to find that some of the intestinal tissues analyzed, such as the ileum and colon, contained barely any detectable MMCs (Figure 1C). Our data indicate that Chm-Cre; Mcl-1^fl/fl^ mice exhibited a significantly reduced number of intestinal MMCs, even when the physiological number of MMCs in wild-type mice was very low (Figure 1C) as previously described (9). MMC populations of the gut are known to expand under inflammatory conditions, such as parasitic infections and allergic conditions (19–21). Further studies will be needed to characterize if and how intestinal MMC numbers change in Chm-Cre; Mcl-1^fl/fl^ mice in these settings. This will help to characterize their role and relevance for intestinal immune responses against parasites and allergens.

Interestingly, we observed markedly reduced MMC numbers in uterine tissue of Chm-Cre; Mcl-1^fl/fl^ mice (Figure 2). Previous studies have shown that uMCs have a positive influence on implantation, placentation, and remodeling of spiral arteries (SAs) as well as placenta size and fetal growth (16, 30, 31). In Chm-Cre; Mcl-1^fl/fl^ mice, the placental and fetal development was largely normal (Figure S1), but we did find that placental thickness is decreased (Figure 3). It has been recently demonstrated that the complete absence of all MCs, i.e., of both CTMCs and MMCs, in *Kit*^*W*−*sh*/^*Kit*^*W*−*sh*^ (sash) mice is linked to a severe impairment in reproduction. The selective depletion of Mcpt-5-positive CTMCs also negatively impacts fetal and placental development (30). Our study suggests that the selective absence of MMCs has no major impact on fetal and placental growth. Previous studies have shown that uterine natural killer cells (uNKs) are able to overcome the absence of uMCs by counterbalancing their effects at the feto-maternal interface to promote SA remodeling and placentation (30). In addition, regulatory T cells (Treg) are known as key regulators of placental implantation. Tregs transferred into abortion-prone mice, which present with insufficient numbers of uMCs, restore SA remodeling and placental development by promoting the expansion of uMCs (16). Further studies will help in understanding the functions of uNKs and Tregs in Chm-Cre; Mcl-1^fl/fl^ mice. uNKs and Tregs may rescue the phenotype in Chm-Cre; Mcl-1^fl/fl^ mice and thereby secure normal implantation and placental development.

Interestingly, we also observed alcian blue/safranin double-positive cells in Chm-Cre; Mcl-1^+/+^ mice (Figure S1). Alcian blue staining is used to detect MMCs, and safranin stains CTMCs. The presence of double-positive MCs in the uterus has previously been reported (32–34). Whether these MCs are in a premature state or in a conversion process is yet unclear. It has been suggested by some authors that uMCs may be able to change their phenotype depending on the surrounding milieu (33, 34).

MCs can originate from bone marrow precursor cells, and they then differentiate into MMCs and CTMCs according to the local microenvironment. BMCMCs are used as an *in vitro* model for studying MC functions. They are a mucosal-like population of MCs since they share some characteristics with MMCs, like their protease composition. The generation of BMCMCs from Chm-Cre; Mcl-1^fl/fl^ mice, however, revealed no differences when compared to Chm-Cre; Mcl-1^+/+^ mice, suggesting that BMCMCs are immature MCs that are phenotypically distinct from both MMCs and CTMCs (35). BMCMCs from Chm-Cre; Mcl-1^fl/fl^ mice were similar in proliferation and maturation as compared to cells derived from control mice (Figures 5B,C), indicating that the hematopoietic progenitor cell capacities for MC differentiation are retained in Chm-Cre; Mcl-1^fl/fl^ mice. This is in line with previous findings showing that the BMCMC population derived from Chm:Cre mice exhibit no specific Cre activity (27), indicating that Cre-expression driven by the baboon α-chymase promotor is restricted to mature MMCs.

After their egress from the bone marrow or alternate stem cell reservoirs, MC progenitors undergo differentiation and maturation in their peripheral target tissues. MC progenitors that populate the skin or peritoneum become CTMCs, whereas those that differentiate and mature in mucosal epithelia and the lamina propria become MMCs. The most important finding of our study is that Chm-Cre; Mcl-1^fl/fl^ mice exhibit a profound reduction of MMCs in mucosal tissues of the glandular stomach and intestine without affecting the number of CTMCs in connective tissues like the skin and the peritoneal cavity.

Although mammalian MCs were first described more than a century ago, their detailed functions remain to be elucidated. MCs are considered to be multifunctional immune cells implicated in several physiological and pathological processes. However, the knowledge about the biological functions of the different MC subtypes, especially MMCs, and the plasticity of MCs is limited due to the lack of suitable models for their investigation *in vivo*. Today, one of the most important challenges for the development of MC-targeting therapeutic applications is to understand their impact on different MC subpopulations. Genetic mouse models are an important tool for this, as well as for the identification and characterization of physiological and pathological functions of MCs *in vivo*. Several MC-deficient models have been described and used in the past: mice deficient for Kit (*Kit*^*W*^*/Kit*^*W*−*v*^ mice and *Kit*^*W*−*sh*^*/Kit*^/*W*−*sh*^ mice), the receptor for stem cell factor (SCF), which is essential for MC growth and survival, have been used for more than 30 years to analyze MC populations and their functions *in vivo*. These mice exhibit a variety of non-MC-related phenotypic abnormalities, including abnormalities affecting hematopoietic cells other than MCs that can contribute to innate or adaptive immune responses, such as neutrophils, basophils, γδ T-cells, and myeloid suppressor cells (9, 11, 36). In recent years, Kit-independent models became available including Cpa3-Cre; Mcl-1^fl/fl^ (28), Cpa3^Cre/+^ (“Cre-Master”) mice (37), and Mas-TRECK (38), as well as Mcpt5-Cre iDTR (39), Mcpt5-Cre R-DTA (40), and RMB mice (41) with an inducible or constitutive deficiency for either the entire MC compartment, CTMCs only, or both MCs and basophils. However, genetic mouse models with a specific focus on MMC populations had not be reported, and this has been a roadblock for improving our understanding of the biology of MMCs.

In this study, we report a novel model for studying MMCs, the Chm-Cre; Mcl-1^fl/fl^ mouse, and we provide a phenotypic characterization of its MC populations. The selective reduction of numbers of MMCs in this novel mouse model is a useful tool in MC research, especially for investigating the role and relevance of MMCs in health and disease.

## Methods

### Mice

Genetically, Chm-Cre; Mcl-1^fl/fl^ mice and congenic normal Chm-Cre; Mcl-1^+/+^ mice (8–12 weeks old) were obtained from breeding colonies of the animal facilities of Charité-Universitätsmedizin Berlin. Mice were kept in community cages at the Animal Care Facilities at light periods of 12 h and were fed water and mouse chow *ad libitum*. All animal care and experimentation was conducted in accordance with current Institutional Animal Care and Use Committee guidelines at the Charité-Universitätsmedizin Berlin under official permissions of the State of Berlin, Germany.

### Development of the Mouse Model

C57BL/6 alpha-chymase-Cre transgenic (Chm-Cre) mice previously described by Müsch et al. (27) were crossed to C57BL/6 Mcl-1^+/fl^ mice for one generation to obtain heterozygote Chm-Cre; Mcl-1^+/fl^ mice. The offspring was identified by PCR genotyping. The heterozygote Chm-Cre; Mcl-1^+/fl^ mice were bred as breeder to obtain Chm-Cre; Mcl-1^+/+^, Chm-Cre; Mcl-1^+/fl^, and Chm-Cre; Mcl-1^fl/fl^ mice.

### Genotyping

Genotyping was performed by PCR. The genotype of transgenic offspring from Chm-Cre; Mcl-1^+/fl^ mice was detected by ear biopsies DNA as described before (22). (Primers: Chm-CreFor: 5′ CGG CGC TAA GGA TGA CTC TGG TCA G 3′, Chm-CreRev: 5′ GTC CAA CGT TCC GTT CGC GCG G 3′, Mcl-1For: 5′ CGA TGC AAC GAG TGA TGA GG 3′, Mcl-1Rev: 5′ GCA TTG CTG TCA CTT GGT CGT 3′). Three reactions are performed during the genotyping, in brief: one to test for Chm-Cre, one to test for the wild-type Mcl-1 allele (PCR product: 360 bp), and one to test for the Mcl-1 flox allele (PCR product: 400 bp).

### Cells

Mouse femoral and tibial BM cells from Chm-Cre; Mcl-1 mice were cultured for 4 weeks in complete IL-3-containing medium with 1% antibiotics to generate BM-derived cultured mast cells (BMCMCs). Peritoneal cells (PMCs) were obtained by injecting Chm-Cre; Mcl-1 mice i.p. with 5 ml of PBS for the peritoneal lavage. Morphological analysis of MCs was assessed by Alkaline-Giemsa staining of cytospins. MC differentiation was assessed by flow cytometric analysis for surface expression of CD117 (c-kit) and FcεRI.

### Histology

Mice were euthanized, and samples of back skin, ear pinna, were fixed in 4% buffered formaldehyde (vendor); stomach, duodenum, ileum, and colon were fixed in 4% methanol-free formaldehyde, dehydrated, and embedded in paraffin ensuring a cross-sectional orientation of all tissues, and 5-μm sections were stained with alkaline-Giemsa solution for histologic examination and enumeration of MCs. To examine stomach MMCs, stomach samples were stained by alcian blue solution. To examine MMCs of the duodenum, jejunum, ileum, and colon, 5-μm sections of intestinal tissue were stained for chloroacetate esterase-positive MMCs, as previously described (35). To examine uMCs, 5-μm paraffin embedded uterus samples were stained by toluidine blue, alcian blue, or safranin. At least three random sections per mouse were analyzed. Ten high-power fields (HPF) per section have been analyzed.

### Determination of MC Numbers

In all histological assessments, cell numbers were enumerated by a single observer not aware of the identity (mouse group) of the individual sections. Cell numbers were based on counting 10 medium-power fields (200×) or HPF (400×), and mean values were calculated. In this study, MCs were classified according to anatomic location as previously described (42). Skin MCs were quantified according to horizontal field length of dermis, uterine MCs were quantified according to area, and gastrointestinal MCs were quantified according to anatomical structure (per field of mucosa and submucosa). MCs superficial to the deep border of the muscular layer, including the epithelium and lamina propria, were classified as MMCs. Images were captured with a Zeiss Axioplan 2 Imaging microscope using a Zeiss AxioCam camera run by AxioVision Rel. 4.8 software.

### Ultrasound Imaging

Serial high-frequency ultrasound measurements were performed with the Vevo® 2100 Imaging system (FujiFilm VisualSonics Inc.) by using the transducer MS550D-0421. Isoflurane (Baxter)-anesthetized mice were placed on the heating platform, abdominal hair was removed with depilatory cream (Reckitt Beckiser), and eye protection cream (Bayer) and prowarmed ultrasound gel (Gello GmbH Geltechnik) were applied. During measurements, ECG, body temperature, and respiratory physiology were controlled. B-Mode was used to visualize anatomical structures in 2D grayscale image. Ultrasound examinations were performed at gd5 (implantation size) and gd10 (implantation size, placenta area/thickness/diameter), and all implantations found within the mothers were imaged. Mice were never exposed longer than 1 h to gaseous anesthesia.

### Statistics

Unless otherwise indicated, all data were tested for statistical significance using the unpaired Student's *t*-test and expressed as mean ± SEM. A *p* ≤ 0.05 was considered to reflect statistical significance.

## Data Availability Statement

The datasets generated for this study are available on request to the corresponding author.

## Ethics Statement

The animal study was reviewed and approved by Landesamt für Soziales und Gesundheit Berlin.

## Author Contributions

YL, QJ, CZ, and NM performed experiments. YL wrote the manuscript. JS and FS supervised experiments and revised the manuscript. AZ, MMe, and MM gave critical input to the experimental design and the manuscript. All authors contributed to the interpretation of the results.

### Conflict of Interest

The authors declare that the research was conducted in the absence of any commercial or financial relationships that could be construed as a potential conflict of interest.

## Supplementary Material

Figure S1 Chm-Cre; Mcl1^fl/fl^ mice exhibit no differences in the analyzed placental parameters comparing to Chm-Cre; Mcl1^+/+^ mice. **(A)** Placental weight of Chm-Cre; Mcl-1^+/+^ (mice *n* = 4, placentas *n* = 21) and Chm-Cre; Mcl-1^fl/fl^ (mice *n* = 5, placentas *n* = 34) females paired with Balb/c males at gd10. **(B)** Number of implantations at gd5 or gd10, abortions, and abortion rate at gd10 from Chm-Cre; Mcl-1^+/+^ (*n* = 3–4) and Chm-Cre; Mcl-1^fl/fl^ (*n* = 3–5) females paired with Balb/c males at gd10. Results are presented as individual values ± median. **(C)** Representative bicorneal uteri of Chm-Cre; Mcl-1^+/+^ and Chm-Cre; Mcl-1^fl/fl^ mice at gd5 or gd10. **(D)** SA wall thickness and SA wall-to-lumen ratio from 3 to 9 SAs per mice of Balb/c-paired Chm-Cre; Mcl-1^+/+^ (*n* = 4) and Chm-Cre; Mcl-1^fl/fl^ (*n* = 5) females at gd10. Statistical differences were analyzed with the Mann–Whitney test. gd, gestation day; SA, spiral artery.
