# Supplementary figures and images for: Chymase-Cre; Mcl-1fl/fl Mice Exhibit Reduced Numbers of Mucosal Mast Cells
Source: Front Immunol. 2019 Oct 15;10:2399. doi: 10.3389/fimmu.2019.02399 (PMC6803453; doi:10.3389/fimmu.2019.02399)

## Figure S1

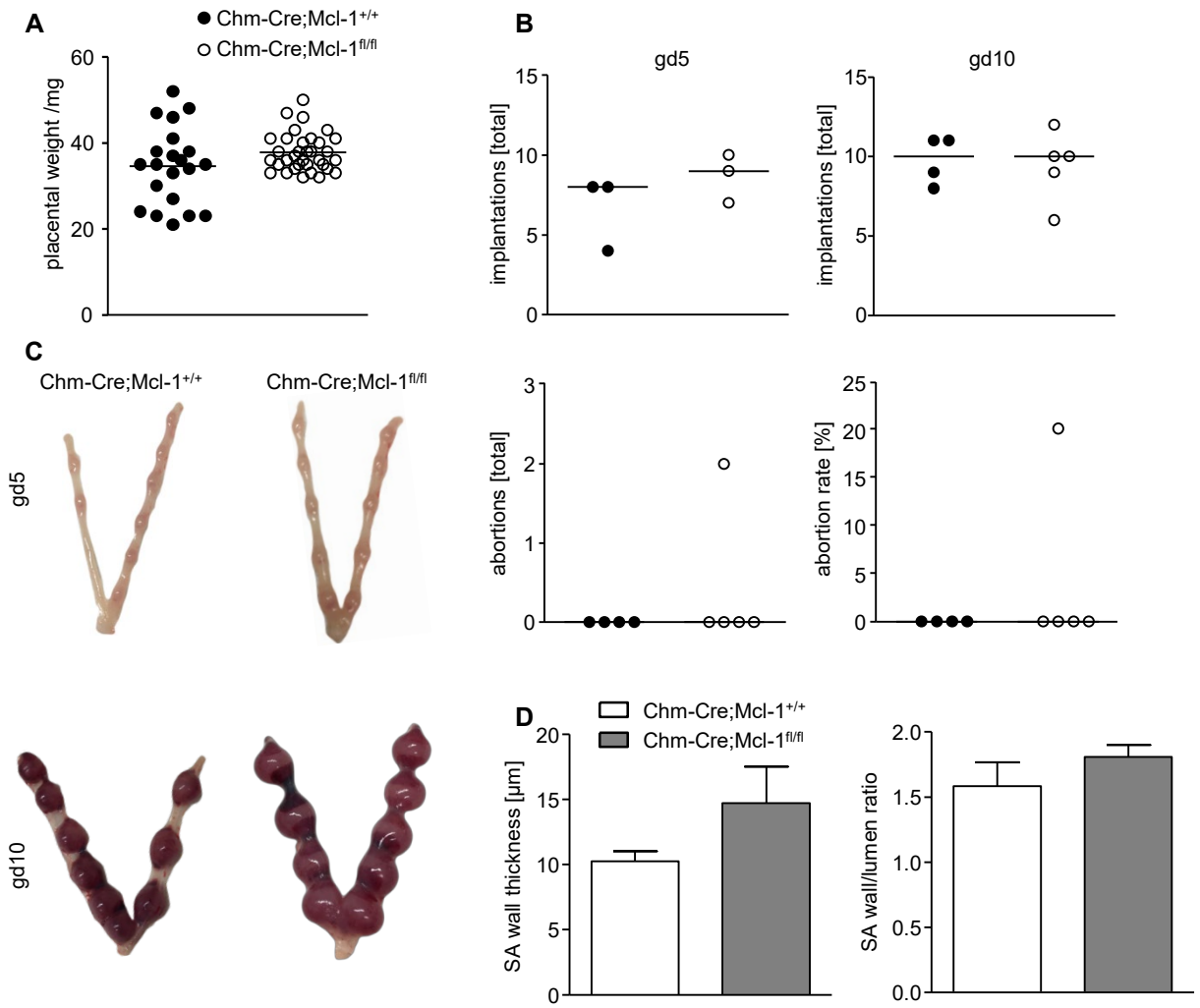

Supplement: Figure S1 — Chm-Cre; Mcl1fl/fl mice exhibit no differences in the analyzed placental parameters comparing to Chm-Cre; Mcl1+/+ mice. (A) Placental weight of Chm-Cre; Mcl-1+/+ (mice n = 4, placentas n = 21) and Chm-Cre; Mcl-1fl/fl (mice n = 5, placentas n = 34) females paired with Balb/c males at gd10. (B) Number of implantations at gd5 or gd10, abortions, and abortion rate at gd10 from Chm-Cre; Mcl-1+/+ (n = 3–4) and Chm-Cre; Mcl-1fl/fl (n = 3–5) females paired with Balb/c males at gd10. Results are presented as individual values ± median. (C) Representative bicorneal uteri of Chm-Cre; Mcl-1+/+ and Chm-Cre; Mcl-1fl/fl mice at gd5 or gd10. (D) SA wall thickness and SA wall-to-lumen ratio from 3 to 9 SAs per mice of Balb/c-paired Chm-Cre; Mcl-1+/+ (n = 4) and Chm-Cre; Mcl-1fl/fl (n = 5) females at gd10. Statistical differences were analyzed with the Mann–Whitney test. gd, gestation day; SA, spiral artery. [file Image_1.pdf]
